# Peer Review of “Challenges in Implementing a Mobile AI Chatbot Intervention for Depression Among Youth on Psychiatric Waiting Lists: Randomized Controlled Study Termination Report”

**DOI:** 10.2196/82074

**Published:** 2025-09-05

**Authors:** Beatrice Tosti

**Affiliations:** 1 University of Cassino and Southern Lazio, Cassino, Italy

**Keywords:** randomized controlled trial, AI chatbot, acceptance and commitment therapy, mental health, psychiatry, children, adolescents, Japan

*This is a peer-review report for “Challenges in Implementing a Mobile AI Chatbot Intervention for Depression Among Youth on Psychiatric Waiting Lists: Randomized Controlled Study Termination Report.”*

## Round 1 Review

### General Comments

The topic and objectives of the study [1] are certainly interesting, as depression among young individuals is an increasingly pervasive and growing problem globally, exacerbated by the COVID-19 pandemic, as the authors themselves point out. Furthermore, the use of artificial intelligence to support traditional methods of treating this condition makes the study topical. The paper is well-written and comprehensible throughout; the supporting bibliography is adequate; it has a good methodological approach, with clear and well-defined objectives, and an accurate description of the inclusion and exclusion criteria for participants. Although the statistical analyses planned by the authors are consistent with the objectives they have defined, the lack of availability of data on which to carry out these analyses and, therefore, the absence of results does not allow an evaluation of this specific aspect. However, the authors have posited potential explanations for instances of nonadherence to the intervention protocol, which are substantiated by extant literature on the subject, therefore apprising the reader of the possible limitations of this type of intervention in this specific population that fulfills certain inclusion criteria. The paper thus provides a cue and guidance for future studies in this field. Lastly, as stated in the major comments below, the major shortcoming of this study is the lack of clarity as to whether the authors used an active or nonactive control group.

### Specific Comments

#### Major Comments

1. In the Study Design paragraph, the authors stated that the control group would receive standard care (making it an active control group), while in the Control Group paragraph, they stated that they would receive general mental health information and would undergo online evaluations and diary recordings (making it a nonactive control group). It is not clear if the authors deem these two procedures similar. In the event that they do not regard them as analogous, it would be beneficial to ascertain which of the two would have been delivered to the control group. Furthermore, it would be appreciated if the authors could provide an explanation and make the appropriate adjustments in the manuscript about (1) what standard care would have comprised and (2) what is the nature of the short video programs that participants received as general mental health information, in order to enable the reader to ascertain whether they are informational videos, mental health support videos, etc.

## Round 2 Review

### General Comments

I would like to express my gratitude to the authors for implementing the requested revisions, which have served to enhance the clarity and thoroughness of the manuscript. Still, there are some elements that, in my view, would benefit from modification.

### Specific Comments

#### Major Comments

1. Supplementary Table 1 and the supplementary figure are missing.
2. The sentence “AI chatbot emol features a friendly character name ‘Roku’” is redundant, as the same concept is repeated in the preceding sentence (in the AI Chatbot Selection Process paragraph).
3. The following sentence is repeated twice: “Weekly online assessments were conducted at Week 0, during the intervention period, and at Week 9” (in the Intervention Group paragraph).
4. The sentence “Non physician research assistants encouraged participants to use the pen consistently for their diary entries and performed minimal mental status checks during these assessments” is redundant, as the same concept is repeated afterward in the same paragraph (Intervention Group section). Therefore, it should be deleted to streamline the text.
5. In what manner was the viewing of the videos organized for the control group? Was a schedule in place, or were the participants free to watch the videos at their own discretion? Furthermore, how was the actual viewing of the videos ascertained?
6. In my personal view, the use of an active control group would have been a valuable approach, for instance, by comparing two distinct chatbots providing different types of therapy, the evaluation of which would have determined which one would prove to be more efficacious in terms of symptoms improvement. This approach would have ensured that both groups received a therapeutic intervention and could have provided additional information in terms of engagement and usability. The authors stated that the design they chose “reflects the real-world experience of many psychiatric waiting list patients in Japan,” but as they also declared, “the lack of timely intervention can exacerbate symptoms and increase the risk of severe outcomes.” Therefore, given such a risk, my question is: what is the rationale behind the authors’ decision to employ a passive control group?
7. The concept expressed in the sentence “Another patient refused participation due to concerns about the diary entry, and the third patient was excluded after starting therapy at another facility” is also conveyed in the preceding sentence (in the Results paragraph). It is recommended that one of the two sentences be deleted.
